# Dignity over DOT: Call to transition from directly observed therapy to rights-based, person-centered care and treatment support

**DOI:** 10.1371/journal.pgph.0005665

**Published:** 2025-12-26

**Authors:** Luan Nguyen Quang Vo, Ashna Ashesh, Yassen Tcholakov, Handaa Enkh-Amgalan, Helene-Mari van der Westhuizen, members of the WHO Civil Society Task Force on Tuberculosis

**Affiliations:** 1 Friends for International TB Relief, Ha Noi, Viet Nam; 2 Department of Global Public Health, Karolinska Institutet, Stockholm, Sweden; 3 Survivors Against TB, Delhi, India; 4 Department of Global and Public Health, McGill University, Montreal, Canada; 5 Stop TB Canada, Ottawa, Canada; 6 TB Advocate and Book Author, Stigmatized (2021), Berlin, Germany,; 7 TB Proof, Cape Town, South Africa; 8 Nuffield Department of Medicine, Oxford University, Oxford, United Kingdom; PLOS: Public Library of Science, UNITED STATES OF AMERICA

## Background

On 23 April 1993, the World Health Organization (WHO) officially declared Tuberculosis (TB) a global public health emergency. In response, WHO introduced a TB policy package branded as the Directly Observed Treatment, Short-course (DOTS) Strategy. To many policymakers, DOTS represents “a multi-component approach that includes directly observed treatment.” [1] However, to most patients, DOTS more closely reflects the innate description that “a health worker must always be present to observe the patient taking the medicines” [2], i.e., directly observed therapy (DOT).

**Fig 1:**
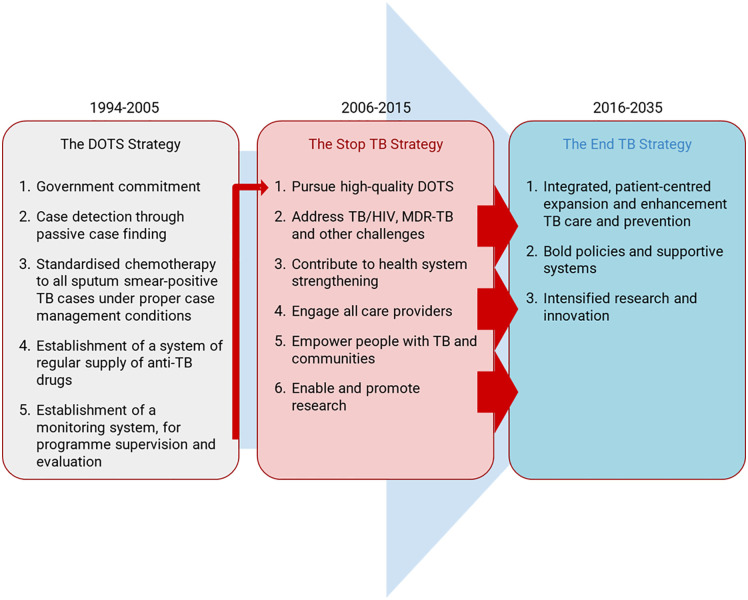
Evolution of global anti-tuberculosis strategies [3]. Notes: DOTS: directly observed therapy, short course; MDR: multidrug resistant. Reproduced with permission of the © ERS 2025.

As strategies progressed from DOTS to Stop TB and now End TB (Fig 1), so did the emphasis on social determinants, gender, rights and universal health coverage (UHC). [4] In 2017, WHO recommended health education and counselling, digital adherence technology (DAT), and flexible variations of DOT, while replacing the DOT nomenclature with treatment support. [5,6] Yet, many National TB Programmes (NTP) have yet to fully adopted this guidance as DOT remains the prevailing tenet of TB care. The COVID-19 pandemic amplified the burden of facility-based DOT on persons with TB and magnified its criticisms in the age of rights-based, person-centered care. [7]

Despite WHO’s emphasis on a person-centered care approach, DOT continues to be a standard feature of TB care in many NTPs. In this commentary, we join the many grassroots campaigns in calling for promoting dignity over disempowerment in TB treatment support. [8,9] Patient surveillance-based mechanisms such as DOT need to make way for person-centered solutions that address the challenges faced by people with TB. WHO guidelines on TB care and support, ethics of implementing the End TB strategy, and social protection for people affected by TB underscore this and offer effective alternatives to compulsory implementation of DOT for people with TB. [10,11]

## Moving from surveillance to rights-based TB care

Evidence suggests that a patient-provider relationship grounded in mutual trust leads to better treatment outcomes (Box 1). This includes promotion of health literacy and education, counseling and provision of reliable information based on solidarity, ethics and rights, so patients feel supported in their arduous journey to recovery. However, when health systems adopt clinical paradigms and render a patient as passive recipient rather than a co-producer of care, this relationship remains inequitable. A prime example of this inequity is DOT.

**Box 1 Perspective from civil society leader and XDR-TB survivor Phumeza Tisile.**

“*My concern with DOT is that there is no trust, already it starts with the assumption that the person won’t take their medication. To me, it was never explained why you have to be observed while taking your treatment. I took this personally. The nursing staff started with a weekly package at first, and once they felt they ‘trusted’ me, they switched to a monthly package. People go to clinics for help for different chronic diseases, so why do we need observation for TB? Having to go to the clinic every day for DOT only adds to the stigma surrounding TB. I call on the broader TB community to take a comprehensive approach to support instead.”*

Intended to benefit both patients and society, DOT has cost people with TB their dignity and agency. The mandate of taking medication under surveillance violates the sense of self and right to autonomy of people affected by TB. The patient is reduced to a potential “defaulter” whose non-compliance is to spite the good intentions of the care provider; their presence is treated as a source of transmission and a threat to society; their right to person-centered care is reduced to empty rhetoric.

DOT further impoverishes TB-affected people, undermining the social outcomes of TB care programs. Instead of addressing the social determinants of TB impacting treatment adherence, relying on DOT perpetuates the disempowering, oppressive and paternalistic approaches to healthcare that sustain the TB epidemic. It reinforces a systemic disparagement of persons affected by TB deemed incapable of managing their own health. DOT has often also been unequally applied between populations often putting a greater strain on societally marginalized groups. This institutionalized paternalism and inequity risks alienating communities and individual care partners, i.e., the patients, to the extent that they may no longer place their trust in the health system and healthcare provider. Further, this adversely impacts health-seeking behavior, gradually drives TB underground, and invariably impedes progress towards TB elimination.

**Fig 2:**
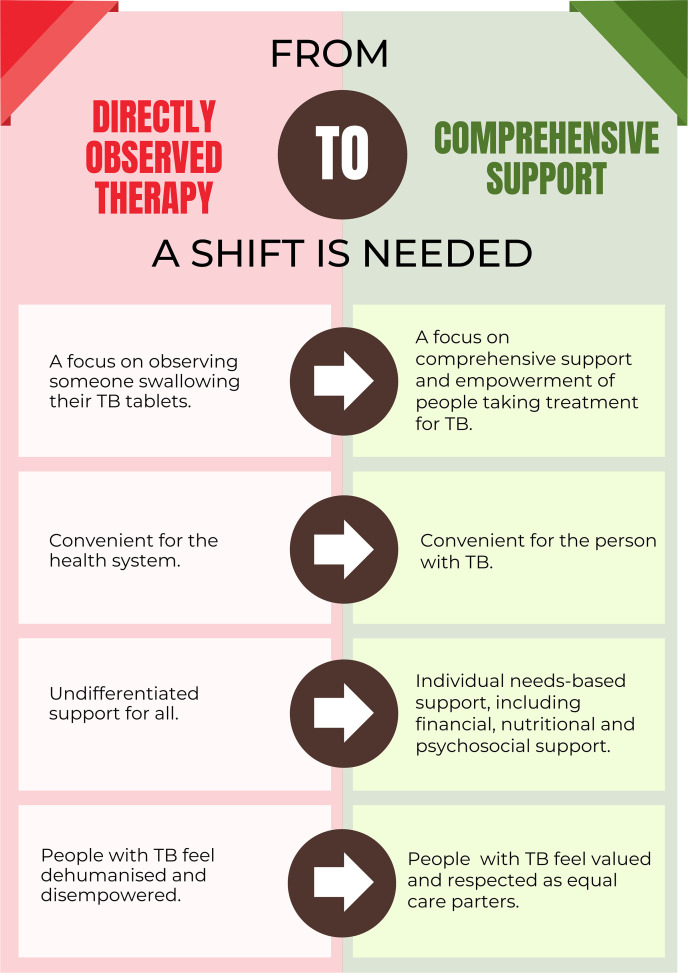
The shifts that are needed.

In critiquing DOT, we do not seek to undermine the importance of successfully completing TB treatment. Our concern lies with the obsolete methods employed to achieve this goal, and how we can shift our current paradigm to TB care to reflect 21^st^ century standards (Fig 2). How can we support patients to complete treatment while preserving their right to autonomy and dignity? Can we have a menu of treatment support options, where patients in consultation with their doctor choose what helps them complete their treatment, thereby empowering them to take ownership of their health?

## Transitioning resources from DOT towards person-centered care

The recognized link between TB and poverty is reflected in the WHO’s End TB Strategy which introduced the catastrophic cost indicator as a performance measure in the fight against TB. Ending TB requires protecting TB-affected households from losing more than one-fifth of their annual household income due to TB. This ambition is in stark contrast, however, with results from 20 national patient cost surveys: an average of 47% catastrophic cost incurrence among TB-affected families. Critically, treatment support such as DOT was associated with 1.5x higher risk of suffering catastrophic cost. [12]

There are major resource implications of sustaining DOT, but can we still record the same marginal gains on clinical outcomes and prevention of drug-resistance that have warranted this heavy-handed approach in the past or are there more patient-centered approaches? The ASCENT consortium hypothesizes that shifting to Digital Adherence Technologies (DAT) is one more cost-effective, equitable way. [13] Yet whether DAT truly constitutes a different approach to DOT is contested by many TB activists, as it is still rooted in surveillance. Seminal studies such as the HRESIPT and RATIONS trials took a step further. These trials aimed to improve treatment adherence and clinical outcomes through intrinsic motivation and social responsibility, thus also meeting the objectives of greater health equity and person-centered care. [14,15] Perhaps it is indeed time to weigh the cost and benefits of DOT, not against self-administered therapy as was popularized in the heyday of DOTS, but against more progressive, person-centered contemporaneous comparators.

## Conclusion

There has been significant progress in the response to the TB epidemic over the past 30 years, evolving from controlling to stopping to finally aspiring to end TB. While we have largely abandoned involuntary care models, a paternalistic remnant is DOT. Still used in many settings, it causes substantial costs to TB-affected people and health systems, and infringes upon the dignity of millions. The era of DOT needs to end. Ethics and protection of human rights must be embraced in practice as an indisputable ingredient of the TB response and the fundamental basis for ending TB. Echoing the political declaration of the United Nations General Assembly High Level meeting on TB, we now call on countries and TB stakeholders to step up their commitment to End TB by building capacity of health staff, involving sectors beyond health and reorienting the health system towards a Primary healthcare-based approach with the community as an equal partner, and to effectively translate WHO recommendations into national policies, action and results to protect the dignity and rights of people with TB (Box 2). Simultaneously, WHO, NTPs and the global TB community must continue to drive the person-centered care agenda and apply an equity, ethics and human rights lens for all policy and technology innovations. With the rapidly evolving evidence base on how to effectively implement person-centered and rights-based TB care and support, we can repurpose the resources for DOT towards more effective and equitable approaches. This way, we can finally move away from a basis of fear to a foundation of trust between patient, provider, health system and the global TB community. Surveillance is the antithesis of trust. It is time to offer patients better treatment support instead of treatment surveillance.

**Box 2 Perspective from Dr Tereza Kasaeva, Director, Department for HIV, Tuberculosis, Hepatitis and Sexually Transmitted Infections, World Health Organization**

“*The World Health Organization has been progressively updating its strategies and policies for tuberculosis prevention and care in line with the emerging evidence and innovations. Since the introduction of the first TB strategies in the 1990s, substantial progress has been achieved toward integrated, people-centered care and prevention, as emphasized in the first pillar of the End TB Strategy. The latest WHO policies recommend people/patient-centered adherence support and the use of approaches, including digital technologies, tailored to each individual’s circumstances, thereby improving treatment outcomes while respecting patients’ rights and preferences. WHO calls for a comprehensive, multisectoral TB response that includes strong community engagement to ensure equitable access to quality care, to address the social determinants and drivers of the TB epidemic, reduce stigma and discrimination, and ensure that no one is left behind”.*
